# Mesenchymal Stem Cells Based Treatment in Dental Medicine: A Narrative Review

**DOI:** 10.3390/ijms23031662

**Published:** 2022-01-31

**Authors:** Igor Smojver, Ivan Katalinić, Roko Bjelica, Dragana Gabrić, Vid Matišić, Vilim Molnar, Dragan Primorac

**Affiliations:** 1 St. Catherine Specialty Hospital, 10000 Zagreb, Croatia; igor.smojver@svkatarina.hr (I.S.); ivan.katalinic@svkatarina.hr (I.K.); rbjelica@sfzg.hr (R.B.); vid.matisic@svkatarina.hr (V.M.); vilim.molnar@svkatarina.hr (V.M.); 2 Department of Oral Surgery, School of Dental Medicine, University of Zagreb, 10000 Zagreb, Croatia; dgabric@sfzg.hr; 3 Eberly College of Science, The Pennsylvania State University, University Park, State College, PA 16802, USA; 4 The Henry C. Lee College of Criminal Justice and Forensic Sciences, University of New Haven, West Haven, CT 06516, USA; 5 Medical School, University of Split, 21000 Split, Croatia; 6 Faculty of Dental Medicine and Health, Josip Juraj Strossmayer University of Osijek, 31000 Osijek, Croatia; 7 Faculty of Medicine, Josip Juraj Strossmayer University of Osijek, 31000 Osijek, Croatia; 8 Medical School, University of Rijeka, 51000 Rijeka, Croatia; 9 Medical School REGIOMED, 96450 Coburg, Germany; 10 Medical School, University of Mostar, 88000 Mostar, Bosnia and Herzegovina

**Keywords:** mesenchymal stem cells, dental stem cells, regenerative dentistry, tissue regeneration, regenerative endodontic treatment, temporomandibular joint disorders

## Abstract

Application of mesenchymal stem cells (MSC) in regenerative therapeutic procedures is becoming an increasingly important topic in medicine. Since the first isolation of dental tissue-derived MSC, there has been an intense investigation on the characteristics and potentials of these cells in regenerative dentistry. Their multidifferentiation potential, self-renewal capacity, and easy accessibility give them a key role in stem cell-based therapy. So far, several different dental stem cell types have been discovered and their potential usage is found in most of the major dental medicine branches. These cells are also researched in multiple fields of medicine for the treatment of degenerative and inflammatory diseases. In this review, we summarized dental MSC sources and analyzed their treatment modalities with particular emphasis on temporomandibular joint osteoarthritis (TMJ OA).

## 1. Introduction

Regenerative dental procedures involving mesenchymal stem cells (MSC) have the potential of becoming a valuable alternative to standard dental treatments that usually rely on the use of artificial materials and/or relatively non-conservative treatments. Major dental branches like endodontics, periodontics, and oral surgery could benefit from the advancement of MSC technology, providing future patients with more conservative treatments with better long-term outcomes [1,2].

Endodontics and restorative dentistry deal with the damaged teeth and inflamed teeth core—the dental pulp. The pulp, responsible for tooth vitality, can be damaged from either bacterial invasion (caries lesion) or trauma. Classic endodontic procedures use chemo-mechanical cleaning of the affected internal tooth anatomy resulting in pulpal tissue loss and obturation of the root canals with synthetic materials, leaving a dead tooth. A non-vital tooth cannot fight infections and is more prone to mechanical damage during normal masticatory function [3]. There have been attempts to explore regenerative treatments and apply MSCs with the purpose of regenerating the pulp but with limited success. The obstacles faced in such procedures are high treatment costs, bacterial contamination of the internal tooth anatomy, and unpredictability of the treatment [4,5].

Periodontal dental medicine (periodontics) battles with oral infections have resulted in the loss of soft and hard tissues that support the tooth, finally leading to tooth loss. Standard treatments, as well as in endodontics, rely on the chemo-mechanical cleaning of the infected area and, if possible, replacing the defects with autologous or synthetic materials with more or less success. The development of MSC dental treatments could help to regenerate lost tissues and prevent further damage to the teeth [1].

The temporomandibular joint (TMJ) is a bilateral diarthrodial joint that participates in important functions such as chewing, swallowing, and speaking. TMJ disorders and conditions (TMD), such as osteoarthritis (OA), lead to cartilage degeneration, disrupted subchondral bone remodeling, and synovitis. The results are chronic pain and reduction of masticatory function, impairing the overall quality of life. Standard conservative treatments including medications, photobiomodulation (BPT) with laser devices, occlusal splints, and extensive prosthodontic rehabilitations or intra-articular injections are used in less advanced TMD stages, while radical surgical methods such as open joint surgeries are favored in severe cases. Such treatments are unfortunately associated with higher risks of complications and often fail to offer a permanent recovery of affected tissues and structures [6].

MSC is currently in the prime focus of researchers because of the evident anti-inflammatory and tissue-regenerating effects. MSCs can be applied both systematically by intravenous or intraarterial application, or they can be delivered locally into the target tissue. Both methods of application follow the same concept; by secreting paracrine molecules (prostaglandin E2, transforming growth factor β1 (TGF-β1), hepatocyte growth factor (HGF), stromal cell-derived factor 1 (SDF-1), nitrous oxide (NO), indoleamine 2,3-dioxygenase (IDO), IL-4, IL-6, IL-10, IL-1 receptor antagonist (IL-1Ra), and soluble tumor necrosis factor-a receptor (sTNFR)), when they are found in an inflammatory environment, MSCs balance the inflammation and enable regenerative processes to take place [7,8]. MSCs promote regenerative processes by secreting anti-scarring (KGF, SDF1, MIP1a, MIP1b), anti-apoptotic (STC-1, SFRP2, TGFB1, HGF), angiogenic (VEGF, TGFB1), mitogenic (TGF-a, TGF-B, HGF, IGF-1, FGF-2, EGF) and antibiotic (LL-37) factors supporting other repair cells [9]. Studies investigating these effects are conducted in multiple fields of medicine for the treatment of degenerative and inflammatory diseases, also including COVID-19 pneumonia and ARDS [10,11,12]. However, their path to daily clinical use is still in its early stages, mainly due to the methodological differences in the literature [13].

The available scientific literature is relatively lacking both in vitro/ex vitro studies and review articles on MSC usage in dental procedures. Thus, in this review of contemporary MSC treatments in dentistry, the current regenerative practices and studies in the field of regenerative dentistry are discussed.

## 2. Dental Tissue-Derived MSC

It is precisely the fact that MSCs can be isolated from dental tissues that has made major progress not only in regenerative dentistry, but in entire regenerative medicine [14]. They can be harvested from teeth and adjacent structures in a non-invasive manner, making them an easily accessible source [15]. Various characteristics of different dental tissue-derived MSCs, such as specific surface antigens, immunomodulatory properties, and main differentiations are shown in Table 1 and thoroughly described in the following subheadings.

### 2.1. Dental Pulp Stem Cells (DPSC)

Two decades ago, the first population of dental MSCs was isolated from the dental pulp tissue of impacted third molars by Gronthos et al. [16]. These adherent cells possess morphology like fibroblasts and exhibit MSC properties [17]. Their high proliferation capacity and multi-lineage differentiation potential give them a privileged status, and they are considered to be an important source of cells in regenerative medicine [18]. DPSCs are positive for surface antigens such as CD13, CD29, CD44, CD59, CD73, CD90, CD105, CD146, and STRO-1, equally to mesenchymal stem cells. On the other hand, they are not positive for surface antigens such as CD14, CD19, CD24, CD34, and CD45, which are hematopoietic stem cell markers [19]. There is data on their odontogenic, angiogenic, myogenic, adipogenic, osteogenic, and neurogenic differentiation potential [17]. The odontogenic differentiation potential of hDPSCs is documented in multiple studies [20,21]. DPSCs generated dentin-pulp-like tissues both in vivo and in vitro [22]. Given their neural crest origin, DPSCs show superior neurogenic potential compared with BMMSCs [23]. Some studies have documented the angiogenic potential of DPSCs, i.e., their ability to differentiate into endothelial cells [24]. They were found to generate visible blood vessels in three-dimensional-printed HA constructs [24]. Taking into consideration the mere structure of dental pulp tissue, it is obvious that the capabilities of neurogenic and angiogenic differentiation make a great contribution to the whole pulp regeneration. Implantation of DPSCs into pulpectomized teeth generated a 3D pulp tissue with vascular and nerve reconstruction [25]. The ability of DPSCs to differentiate into osteoblasts and contribute to bone regeneration has also been reported. They were found to express some of the typical osteoblastic markers, such as alkaline phosphatase (ALP), osteopontin (OPN), and osteocalcin (OCN) [22]. The potential benefits of DPSCs use in bone tissue engineering are thoroughly described in a systematic review by Leyendecker et al. [26], concluding that DPSCs are one of the most promising sources of MSCs for the reconstruction of bone defects. Aside from the multi-lineage differentiation potential of DPSCs, they also exhibit immunomodulatory properties. Anti-inflammatory cytokines (transforming growth factor-beta (TGF-β), prostaglandin E2 (PGE2), and interleukin-6 (IL-6)) are released from DPSCs [27]. Kwack et al. found that DPSCs are responsible for the inhibition of acute allogeneic immune responses by stimulating T cells to release TGF-β [28].

### 2.2. Stem Cells from Exfoliated Deciduous Teeth (SHED)

The pulp of human exfoliated deciduous teeth is another source of dental stem cells. Stem cells from exfoliated deciduous teeth (SHEDs) were firstly obtained from the pulp of exfoliated deciduous teeth by Miura et al. [29]. SHEDs are also named “immature DPSCs” due to the immature cell population in exfoliated deciduous teeth [30]. There are some differences between SHEDs and DPSCs, such as higher proliferation ability, sphere-like cluster formation, and more cell-population doublings [29]. SHEDs are positive for surface antigens CD166, CD146, CD90, CD73, CD29, which are mesenchymal stem cell markers, while on the contrary, they are negative for CD45, CD34, and CD14 [31]. Gene expression of SHED cells differs from that of DPSCs. Nakamura et al. observed higher expression in SHED for genes that participate in pathways related to cell proliferation and extracellular matrix, including several cytokines such as fibroblast growth factor (FGF) and TGF-β [32]. SHEDs are capable of giving rise to various lineages of cells, such as osteocytes, chondrocytes, adipocytes, odontoblasts, endothelial cells, and neuron-like cells [33]. Their capability to differentiate into odontoblasts and endothelial cells, consequently forming dentin-like and pulp-like tissue, was observed in mice [34]. Furthermore, while cultured under neural inductive conditions, SHEDs were able to differentiate into neural cells and presented higher expression levels of neuronal and glial cell markers, such as β-III-tubulin, tyrosine-hydroxylase (TH), microtubule-associated protein 2 (MAP2), and Nestin than DPSCs [33]. As for osteogenic potential, SHEDs are recorded to induce new bone formation by recruiting host osteogenic cells in vivo, rather than differentiating into osteoblasts which happened in vitro [17]. Apart from the differentiation abilities written above, SHEDs also demonstrate immunomodulatory functions. They play an important role in the defense against microorganisms by repressing the function of T helper 17 (Th17) lymphocytes [35]. Local delivery of SHEDs increased the number of CD206^+^ M2 macrophages in periodontal tissues, thus attributed to the reduction of periodontal tissue inflammation and enhancement of periodontal regeneration [36].

### 2.3. Stem Cells from Apical Papilla (SCAP)

Stem cells from apical papilla (SCAP) were first discovered and isolated from the apical papilla tissue of incompletely developed teeth by Sonoyama et al. in 2006 [37]. The apical papilla is loosely attached to the apices of immature permanent teeth and is different from the pulp in terms of containing fewer cellular and vascular components than the pulp tissue [38]. These stem cells are characterized by high proliferative potential, self-renewal ability, and low immunogenicity. Thus, SCAPs are capable of giving rise to various lineages of cells, including osteogenic, odontogenic, neurogenic, adipogenic, and chondrogenic cells, which gives them an important role in regenerative dentistry [39]. It is documented that SCAPs exhibit a higher proliferation rate than DPSCs and periodontal ligament stem cells (PDLSCs), but conversely, lower proliferation rate than dental follicle stem cells (DFSCs) [40]. Furthermore, SCAPs have greater migration ability assessed by scratch assay than DPSCs [37]. They express surface antigens specific for mesenchymal stem cells such as CD146, CD90, CD44, CD24, and STRO-1. On the contrary, they do not express surface antigens specific for hematopoietic stem cells [2]. It is worth mentioning that CD24, which is not perceptible in BMMSCs and DPSCs, may be used to distinguish SCAPs from these cells [39]. Studies have confirmed that SCAPs are able to differentiate into odontoblasts and osteoblasts [37,38,41]. They express specific markers of osteoblasts or odontoblasts, such as alkaline phosphatase, runt-related transcription factor 2, osteocalcin, dentin sialophosphoprotein, bone sialoprotein, and dentin matrix protein 1 [39]. Since SCAPs are neural crest-derived cells, there is in vitro evidence of their neurogenic differentiation capacity after induction [41]. Moreover, forming adipocytes after induction with adipogenic medium or cartilage identified by alcian blue staining under appropriate culture conditions definitely identifies SCAPs as cells of immensely high proliferation potential [40,42]. Ding et al. documented suppression of T cell proliferation by SCAPs in vitro through an apoptosis-independent mechanism, which makes them a potential immunotherapeutic tool [43].

### 2.4. Periodontal Ligament Stem Cells (PDLSC)

Periodontal ligament provides the connection between alveolar bone and cementum, containing progenitor cells that can maintain tissue homeostasis and regeneration of periodontal tissues. These cells also display mesenchymal stem cells characteristics [22]. Similar to other dental stem cells, they express surface antigens specific for mesenchymal stem cells like CD105, CD73, CD44, CD29, and CD10, but not those specific for hematopoietic stem cells like CD14, CD34, and CD45 [17]. The multidifferentiation potential of PDLSCs is noticeable through their capability to differentiate into chondrogenic, osteogenic, neurogenic, and adipogenic cells [22]. PDLSCs have been reported to format calcified nodules and express ALP, matrix extracellular protein (MEPE), BSP, OCN, and TGF-β receptor I, which shows their cementogenic/osteogenic differentiation potential [44]. In the same study, PDLSCs were transplanted into the rat periodontal lesion sites and generated typical PDL/cementum-like structures. Together with SCAPs, PDLSCs also contribute to root regeneration. Sonoyama et al. transplanted both human SCAP and PDLSCs in a minipig to generate a root/periodontal complex capable of supporting a porcelain crown [37]. There is evidence of osteogenic and bone regeneration properties of extracellular vesicles (EVs) released by PDLSCs. PDLSCs—EV, together with collagen membranes, were transplanted into bone defects of rats and showed osteoid formation with an osteoblast-like structure in implant sites [45]. It is worth mentioning that high therapeutic concentrations (>1.5 μM) of zoledronic acid, which is a nitrogen-containing bisphosphonate drug (N-BP), impair the viability, induce apoptosis and decrease osteogenic differentiation of PDLSCs [46]. Furthermore, PDLSCs also spontaneously express neural protein markers such as nestin and growth-associated protein-43 (GAP-43), leading the way to the potential use of these cells in cell-based therapy in neurodegenerative diseases [47]. The immunosuppressive ability of PDLSCs was documented in several studies [48,49]. PDLSCs are the only dental tissue-derived MSCs showing cyclic stretch-induced exosome secretion and are responsible for the suppression of IL-1β production via the inhibition of NF-κB signaling pathway [50]. Peripheral blood mononuclear cells (PBMNCs) are known for their vital role in the innate and adaptive immune response, and PDLSCs are reported to suppress their proliferation [51]. Nagata et al. [52] also supported the fact of immunosuppressive nature of PDLSCs in their study. Transplantation of conditioned medium of PDLSCs resulted in the decreased mRNA level of tumor necrosis factor-α (TNF-α) in healing periodontal tissues, thus suppressing the inflammatory response.

### 2.5. Alveolar Bone-Derived Mesenchymal Stem Cells (ABMSC)

Alveolar bone is embryonically derived from the dental follicle and resembles a thickened ridge containing the tooth sockets that hold teeth [49]. Collecting alveolar Bone-derived mesenchymal stem cells (ABMSCs) from alveolar bone during the course of dental surgery is a favorable isolation method [53]. ABMSCs are found to have favorable osteogenic differentiation potential comparable to BMMSCs, but their potential to differentiate into chondrocytes or adipocytes is weaker [53]. They express the surface markers analog to MSCs such as CD73, CD90, CD105, and STRO-1, but do not express the hematopoietic markers CD14, CD34, and CD45 [49]. Wang et al. confirmed the osteogenic differentiation potential of ABMSCs in their study [54]. Transplantation of ABMSCs and porous nano-HA/collagen/PLA scaffold into the critical-sized mandibular bone defect of a rabbit resulted with new bone formation. Furthermore, osteogenic and adipogenic gene expressions were evaluated in vitro by reverse transcription-polymerase chain reaction, and the formation of mineralized nodule and adipocytes was also detected. Upon hABC transplantation in vivo, significant ectopic bone formation was induced with the characteristics of fully matured bone tissue [55]. ABMSCs exhibited immunosuppressive effects on monocyte and T cell activation similar to BMSCs and the protein arrays identified interleukin (IL)-6 and monocyte chemoattractant protein (MCP)-1 to be the major cytokines secreted by ABMSCs. These data suggest that these cells have potent immunomodulatory properties [56].

### 2.6. Gingival-Derived Mesenchymal Stem Cells (GMSC)

Gingival-derived mesenchymal stem cells (GMSCs) are progenitor or stem cells first identified in the spinous layer of the human gingiva. They were shown to exhibit multipotent differentiation and self-renewal capacity, as well as immunomodulatory properties [57]. Gingiva is an easily accessible tissue during routine dental procedures, which makes it a feasible source of stem cells in regenerative dentistry [22]. GMSCs have multipotent mesenchymal precursor cell properties and the ability to differentiate into various cell types, such as chondrocytes, osteoblasts, and adipocytes, as determined by the expression of specific surface antigens [58]. GMSCs were found to form deposits with positive Alizarin Red S staining and upregulated expression of OCN in vitro, indicating their osteogenic differentiation [57]. In a recent study, EVs derived from GMSCs revealed high expression levels of RUNX2, bone morphogenic protein (BMP) 2 and 4, and abundant ECM and nodules of new bone formation, which confirms their significant osteogenic properties [59]. Transplantation of GMSCs resulted in the formation of connective-like tissues expressing collagen I, which is absent in PDLSCs [57]. GMSCs contain precursor cells to differentiate into gingival cells, thus having an ability for gingival differentiation automatically in vivo. After transplantation of human GMSCs into gingival defects of rats, new normal-like gingival tissue was generated [60]. Since GMSC spheroids have shown differentiation potential into both neuronal and Schwann-like cells, they are considered an up-and-coming potential for nerve regeneration and functional recovery. 3D bioprinted grafts with GMSCs formed nerve tissue with complete coverage of segmental defects in rat facial nerves [60]. GMSCs are also known for their immunomodulatory functions. They enhance the secretion of several chemokines and cytokines and improve resistance to oxidative stress-induced apoptosis [61]. The same authors found that 3D spheroid GMSCs could attenuate chemotherapy-induced oral mucositis in the murine model, which correlates with already mentioned resistance to oxidative stress-induced apoptosis.

### 2.7. Dental Follicle Stem Cells (DFSC)

The dental follicle is ectomesenchymally derived connective tissue surrounding the tooth germ prior to eruption [49]. It contains progenitor cells for periodontal ligament cells, cementoblasts, and osteoblasts [22]. These cells are similar to other dental stem cells, therefore, they possess a substantial proliferative ability, express similar cell surface antigens and are capable of forming hard tissue both in vitro and in vivo [62]. DFSCs exhibit higher proliferation potential and colony-forming ability than DPSCs, SHEDs, and PDLSCs, which is crucial for their potential use in regenerative dental medicine [22]. DFSCs show higher expression levels of osteogenic-related markers such as RUNX2 and ALP compared to SHEDs and DPSCs [63]. They are also immature cells with less heterochromatin in the nucleus and fewer organelles in the cytoplasm compared to PDLSCs on ultrastructural comparison [64]. Their potential for periodontal differentiation is observed through their ability to form PDL-like structures or calcified nodules with bone- or cementum-like structures in vitro. Furthermore, in vivo transplantation of DFSCs could produce a cementum/PDL-like complex. This suggests that DFSCs could be a viable source for bio-root engineering [65]. It is documented that DFSCs have the preferable potential for odontogenic differentiation compared to PDLSCs, due to higher expression of dentin sialophosphoprotein (DSPP) [22]. DFSC also generated the entire dentin structure upon the induction of treated dentin matrix (TDM) [45]. Cultured DFSCs stimulated by BMP-2 and -7 and enamel matrix derivatives acquired cementoblast features in vitro [66]. The immunomodulatory effects of DFSCs also favor their potential to treat immune diseases. Production of TGF-β and suppression in the proliferation of PBMCs was caused by DFSCs [67]. In addition, TLR3 and TLR4 agonists augmented the suppressive potential of DFSCs and potentiated TGF-β and IL-6 secretions [49].

### 2.8. Tooth Germ Stem Cells (TGSC)

Tooth germ stem cells (TGSCs) were isolated and identified in the dental mesenchyme of the third molar tooth germ during the late bell stage [49]. They are easily accessible because they are obtained from removed teeth during regular dental procedures [68]. The mesenchymal phenotype of TGSCs is indicated by the expression of MSC-associated markers and pluripotency-associated genes (nanog, oct4, sox2, klf4, c-myc). They express surface markers characteristic for MSC-s such as CD73, CD90, CD105, and CD166, but are negative for CD34, CD45, and CD133. TGSCs can differentiate into osteogenic, adipogenic, and neurogenic cells and also form tube-like structures in Matrigel assay [68]. Their osteogenic differentiation ability has been documented, as the new bone formation was detected in the pore area of the HA/TGSC implants. Furthermore, chondrogenic differentiation capability was confirmed by the expression of hyaline cartilage-specific extracellular matrix (ECM) and type II collagen after TGSCS attachment to 3D biological scaffolds [69]. BMP-2 and BMP-7 can be transferred into TGSCs by electroporation, thus increasing the odontogenic and osteogenic differentiation abilities of TGSCs [70]. It implies that TGSCs are used for gene therapy applications. It is of crucial importance that these cells express important transcription factors that could render TGSCs an attractive candidate for future somatic cell re-programming studies to differentiate tooth germs into various tissue types [68].

## 3. Stem Cell Banking in Dental Medicine

Although the isolation of dental tissue-derived stem cells is less invasive and easier to obtain than it is with other stem cells, long-term culture of these cells goes along with unpropitious effects, including contamination, phenotypic instability, and cell death [71]. Banks specializing in collecting bone marrow or placental cord have existed for decades, but dental stem cells banks are, unlike them, relatively new. Thus, adequate storage is required to ensure not only the viability of these cells but also their phenotypic stability, multidifferentiation potential, and to protect them from microbiological contamination [72].

A broad variety of protocols regarding the collection of stem cells exists. The protocols and technical details of the entire process of stem cell banking are thoroughly described in a recent review written by Khaseb et al. [73]. However, regardless of the method by which the sample was obtained, storage for transport has a key role in cell survival [72]. A lot of studies have evaluated the effect of different transport media on the preservation of live teeth [74]. Bovine milk, along with autologous saliva, is still recommended as a carrier for avulsed or extracted teeth. It is biocompatible, naturally buffered, and has a neutral pH. Above all, it is inexpensive and commonly available [75].

Cryopreservation enables stem cells to retain their properties and it is essential for their long-term preservation and storage [76]. Cells are suspended in a preservation medium, which contains a cryoprotectant, most commonly dimethyl sulfoxide (DMSO). DMSO inhibits the growth of ice crystals that may impair cell membrane, thus reducing the viability of stem cells [76]. Potential cytotoxicity of DMSO has prompted researchers to find other strategies to reduce cryoprotectant toxicity or to develop xeno-free solutions [76,77,78]. Besides the importance of preservation medium, careful control of cooling rates is the most important aspect of the process. To slow freezing causes dehydration and osmotic stress-shrinkage, whereas rapid freezing results in intracellular ice crystals formation [76]. The possibility of long-term storage and preservation of stem cells offers extraordinary perspectives for regenerative and translational medicine.

## 4. Regenerative Dentistry

The concept of tissue engineering is generally based on three elements, also known as the tissue engineering triad. Each of them possesses unique biological capabilities, and all of them are of utmost importance in regenerative medicine. The first element of the triad are stem cells with their potential to differentiate into a particular tissue in a suitable medium (scaffold), which is the second element of the triad, under the influence of bioactive molecules or growth factors (third element) [22,79].

### 4.1. Tissue Regeneration Based on Scaffolds

The scaffolds are defined as three-dimensional biocompatible structures whose role is to provide an adequate environment for cells seeded onto their surfaces, to promote cell proliferation and differentiation into lineages [79,80]. According to this, their contribution to the formation of ECM by providing support to cells to adhere, grow and differentiate is undeniable [79]. Due to their multidisciplinary usage, the classification of scaffolds slightly differs among the authors [81]. The most common classification is based on form, origin, size, presence of cells, and degradability of scaffolds [79]. Oral and maxillofacial surgeons are constantly investigating an ideal bone scaffold material, thus, a wide range of polymers, composites, and ceramics have been observed [81]. Studies involve a broad variety of bone defects, from periodontal defects to critical-sized defects [82]. Even though autogenous bone grafts are the gold standard for treatment of critical sized-defects, invasiveness, limited availability, morbidity in donor sites, and secondary surgery represent crucial disadvantages, leading to a search for a more suitable alternative [83]. An ideal scaffold has to be as similar as possible to the natural ECM. It includes biocompatibility, mechanical properties, biodegradability, porous structure, and suitable surface chemistry for cell proliferation [81]. A wide variety of biomaterials are applied for scaffold processing, such as natural or synthetic polymers, ceramics, and hydrogels. Biological and mechanical properties and fabrication techniques need to correspond to the bone engineering strategy to achieve an optimal scaffold performance and predictable outcome of therapy [84]. Simu et al. found that high viscosity soft propolis extract and shell clam-based biomaterial acts as a suitable scaffold, promoting human stem cells attachment, proliferation, differentiation, and presenting an important osteoinductive effect essential for the mineralized tissue reparation process [85]. Scaffolds are also widely used in regenerative endodontic treatment (RET). There are four most frequently used groups of scaffolds in RET: autologous platelet concentrates [APC], nanofibrous scaffolds, injectable scaffolds, and bioactive molecule carrier systems [79]. Several studies confirmed that the use of nanofibers and injectable scaffolds, with or without the presence of stem cells and/or growth factors, for intracanal drug delivery creates a bacterium-free environment, stimulates pulp and dentin regeneration, and is a potential novel therapeutic strategy in endodontic treatment [86,87]. The treatment of chronic oral inflammations, including periodontitis, has also been performed with scaffolds and stem cells [88]. Scaffolds provide a contact guidance enabling migration of cells into periodontal defects, therefore inducing regeneration. However, there is no scaffold system with successful clinical outcomes in treating periodontal defects. It is attributed to the limited understanding of in vivo degradation of implanted scaffold materials [89]. The long-term safety and clinical effectiveness of scaffolds is an unavoidable topic in regenerative dentistry and translational medicine. Issues that affect scaffold use are in situ degradation of materials, infections, and immunologic reactions to bio-scaffolds [80]. Consequently, novel scaffold-free approaches are developed in order to exclude the use of biomaterials and avoid the plausible risks [90]. The development of 3D culture systems without scaffolds such as spheroids, organoids and organ germs are an immense step forward in tissue engineering [91]. A number of these novel strategies were brought into the focus of researchers and seem to have promising applications in regenerative medicine [22,80,92].

### 4.2. Growth Factor Delivery-Based Tissue Regeneration

Growth factors are signal molecules involved in the stimulation of cell proliferation, differentiation, and prevention of apoptosis. The main function of these proteins is the external control of the cell cycle by stimulation of the cells to enter into phase G1 [93]. Growth factors bind to corresponding surface receptors and initiate signal pathways that either activate cytoplasmic proteins or induce transcription of new proteins [94]. They usually have a short half-life and are quickly eliminated. The crucial role of growth factors in tissue regeneration and engineering is based on their ability to determine the fate of stem or progenitor cells [95]. Dentin matrix is a viable source of various biological molecules, including growth factors [96]. Demineralization processes, but also irrigants and medicaments used in RET release these molecules from dentine. Furthermore, these growth factors induce cells mobilized into root canals by apical bleeding to regenerate pulp tissue. Cell migration is induced by fibroblast growth factor 2 (FGF2), platelet-derived growth factor (PDGF) and TGF-β; cell proliferation by TGF-β1, FGF2, vascular endothelial growth factor (VEGF) and insulin-like growth factors (IGFs); angiogenesis by VEGF and dentinogenesis by BMP and FGF2 [97]. There are studies that recorded regeneration of pulp–dentine complex by exogenous growth factors implanted into root canals to contribute to endogenous biological molecules of dentine matrix [98].

A wide range of bone grafting techniques have been developed thanks to growth factors which are considered as the best way to induce tissue regeneration. Several of these molecules have been studied, including PDGF, TGF-β, FGF, VEGF, IGF, and PRP [99]. They play a major part in bone regeneration by stimulation of cell proliferation, angiogenesis, and remodeling of the ECM. Scaffolds are frequently used alongside growth factors to provide support and guide tissue healing [93]. Marx et al. observed greater bone density and radiographic maturation rate of grafts enriched with PRP than control group grafts [100]. PRP is also a source of PDGF and TGF-β. These factors are released from degranulated platelets in the graft and are closely related to the initial phase of bone regeneration [99]. Bone formation in the later healing period was found to be effectively enhanced with PDGF and absorbable collagen sponge as a carrier [101]. The application of angiogenic agents such as VEGF alone or in association with BMP-2 was investigated by Zhang et al. [102]. According to this study, VEGF alone was not sufficient enough for bone generation, but applied together with BMP-2, it significantly improved bone formation. BMPs are proteins classified as the subpopulation of the TGF-β superfamily [103]. Two of them (BMP-2 and BMP-7) play an important role in bone formation and repair [104]. The US Food and Drug Administration approved the use of recombinant human BMP (rhBMP) in combination with a collagen sponge carrier for clinical situations, including sinus augmentation and alveolar ridge augmentation [99]. Osteoconductive and osteoinductive properties of growth factors have already made them a viable factor in regenerative dental medicine, but further development of carriers, a better understanding of pathways, and biology of healing processes is of utmost importance to target the new therapeutic strategies.

### 4.3. Regenerative Endodontic Treatments (RET)

In contrast to conventional treatments, regenerative endodontic treatments (RET) aims to replace, regenerate or restore diseased or traumatized dental tissues and cells. The idea behind RET is not new; it was pioneered by Nygaard-Ostby in 1961 and 1971 [105,106]. These first RET attempts relied on induced bleeding from periapical tissues into the chemo-mechanically cleaned root canal space. In 2001, Iwaya et al. reported treating immature permanent teeth with apical periodontitis and sinus tract. Their paper used the term revascularization, since a histological analysis of the root canal space showed newly formed vascular tissue, important for repair mechanisms [107]. Later in 2007, the term “regenerative endodontics” was adopted by the American Association of Endodontists (AAE) [108]. They defined RET as “biologically based procedures designed to replace damaged structures, including dentin and root structures, as well as cells of the pulp dentin complex”. In 2016 AAE set three goals for the measurement of RET success [109]. First, the resolution of periapical periodontitis and clinical symptoms. Second, a thickening of root walls and/or continued root maturation. Third, positive response to pulp sensibility testing (neurogenesis). In addition, the case selection for RET should focus on permanent teeth with necrotic pulp and immature teeth that do not need an intra-radicular post for the final restoration. The treating patient should be compliant and not have any allergies to medicaments required for the procedure [2].

#### 4.3.1. RET Strategies

**Figure 1:**
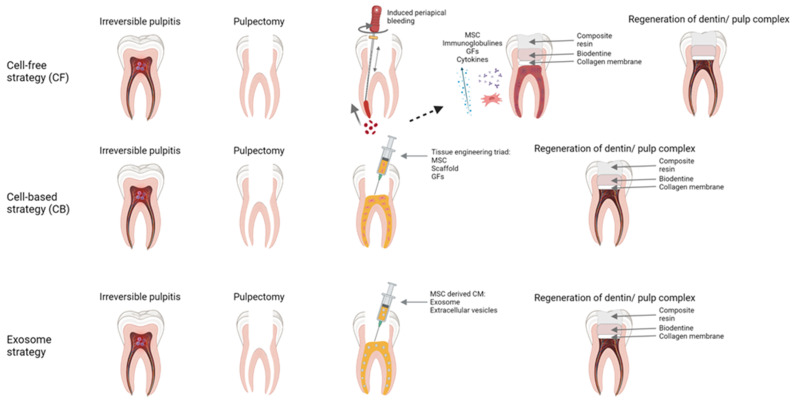
Schematic representation of three different regenerative endodontic treatment (RET) strategies. Cell-free strategy (CF) mainly relies on induced bleeding from periapical tissues, bringing different cells like mesenchymal stem cells (MSCs), immunoglobulins, cytokines, and growth factors (GFs) into a root canal. Cell-based strategy (CB) relies on the interplay between three main factors of the “tissue engineering triad”: stem cells, signaling molecules, and scaffolds. Exosome strategy presents an intermediate approach using conditioned medium (CM) of MSCs. Created with BioRender.com.

It can be stated that the early RET attempts were (and still are) a part of a cell-free strategy (CF) that mainly relies on induced bleeding from periapical tissues (Figure 1). The blood may bring in different cells, including MSCs, immunoglobulins, cytokines, and growth factors [110,111]. The strategy is limited to immature teeth (teeth with incomplete root development) due to several reasons. The induced bleeding brings in the stem cells from the apical papilla [112]. Apical papilla, a valuable source of MSCs that plays a relevant role in root development, is present only in immature teeth [112]. Mature teeth have a smaller amount of available stem cells coming from periapical tissues (bone marrow, periodontal ligament tissue/inflamed tissue), and the apical pathway for cell migration is narrower. These cells have a lower capacity to recreate complex pulp tissue, and their ability to differentiate decreases with age; thus, older patients have lower healing potential [113,114]. Root canal disinfection is also more challenging due to the complex root canal anatomy [112]. Histology of CF attempts mainly shows repair effects and not a real regeneration of pulp–dentin complex [95,115]. Finally, the prognosis of the RET treatments is relatively unpredictable [115]. A cell-based strategy (CB) is a more recent approach to RET, first demonstrated in dogs and later in humans [116]. A CB strategy relies on the interplay between three main factors (the classic tissue engineering triad) exogenously delivered to the previously disinfected and prepared root canal space (Figure 1):

(1) Stem cells. Autologous stem cells are a reliable source since they are harvested from the patient (dental pulp, periodontal ligament, adipose tissue, etc.) [117]. Allogenic stem cells may be more practical and cost-efficient because they are available off the shelf [118].
(2) Signaling molecules. They activate the stem cells (various growth and differentiating factors available from PRF, PRP, or blood clots) [119,120,121].
(3) Scaffolds. A three-dimensional, porous, biodegradable, and biocompatible materials that mechanically supports the cells, allowing efficient nutrient and gas exchange [79,122]. They can be of natural origin (hyaluronic acid, collagen, PRP, PRF, blood clot) or of artificial origin (polymers of polyglycolic acid, glass-ceramic, bioactive glass, etc.) [79,123,124].

Some research shows that the cells prepared for transplantation can be additionally conditioned with the visible light treatment, thus obtaining better final proliferation and differentiation of the cells [125]. Photobiomodulation therapy (PBMT) is well researched and widely accepted method in regenerative medicine and dentistry. It relies on the interaction between the visible light produced by the LED or laser devices, mainly in the red spectrum (660 nm), bearing low-level energy (mW) to the cells. Intracellular chromophores absorb the energy of the light and increase DNA activity, RNA, ATP, and various proteins synthesis [126,127,128]. To conclude, although when applied, the CB show promising effects (recent studies claim post-treatment formation of a vascularized tissue with a normal physiological response-vital pulp-like tissue), they are relatively complex, expensive, and not yet thoroughly explored [129]. There are also no clear ideal strategies, or AAE/EAO recommended protocols [4,5].

#### 4.3.2. Exosome Strategy

This strategy could present an alternative, intermediate approach in between CB and CF strategy. The idea for the strategy is founded on the evidence that conditioned medium of MSCs could have positive effects on ischemic heart disease [130]. Since then, there has been an increase in interest in the use of exosomes instead of MSCs for the purpose of regenerative medicine [131]. In the exosome strategy, everything is the same as in the CB strategy except that MSCs are replaced with MSC-derived exosomes. Exosomes can be defined as extracellular vesicles secreted by the cells. Vesicles exit the cells through the fusion process with the cell membrane and their size range from 40–150 nm in diameter; their content-lipids, proteins, and nucleic acids depend on the source cell type [132]. MSC-derived exosomes are comprised of signaling molecules like interleukins, chemokines, cytokines, growth factors, and miRNA, small single-stranded non-coding RNA molecules [133]. Due to its content, exosomes can modulate the target cells. They are important for cell communication and participate in both pathological and physiological cell processes [134]. According to some studies [135,136,137], MSC-derived exosomes exert similar effects to MSCs. Other cells ingest exosomes by phagocytosis or endocytosis [138]. Vesicles cannot renew themselves and lack the potential for tumorigenesis [139]. Their toxic potential is low due to the fast clearance (no unfavorable liver accumulation), and they do not express class II human leukocyte antigens (HLA), meaning they are hypoimmunogenic [131,140]. There are many exosome harvesting strategies (centrifugation, ultrafiltration, chromatography, etc.) [141]. Following the harvest, exosomes can even be stored or biobanked through the process of dry-freezing, making them more available and more practical for scientific or clinical use [142]. A recent review paper by Ivica et al. focuses on possible exosomes applications in dentistry [5]. The authors state that the evidence for exosome use in regenerative dentistry comes from limited in vitro or animal in vivo studies, so there are currently no sufficient data for safe and practical clinical usage. According to this evidence, “exosomes may have a positive effect on the differentiation of native cells, their migration, proliferation, and angiogenesis”, resulting in the similar pulpal regenerative potential seen in the CB approach, but without technical problems that CB approaches inherently bring along [5]. Since MSC-derived exosomes mimic the effects of MSCs, it can be expected that someday they will be used in clinical regenerative endodontics like MSCs are used today [5]. It is worth mentioning that all the above written approaches of RET treatment are dependent on the regenerative capacities of dental stem cells [143]. Thus, the development of novel strategies integrating signaling pathways and gene expression that could contribute to the pulp regeneration is of crucial importance. To conclude, different RET treatments and strategies (Figure 1) show a huge potential to become a valuable treatment modality in contemporary endodontics, but more technical advances, researches and clinical studies are needed to firmly establish practical, economical, reliable and predictable clinical protocols.

### 4.4. MSC Based Regenerative Periodontal Treatments

Periodontal dental medicine (periodontics) battles with infections of the periodontal tissues (periodontitis). Periodontal infections cause loss of soft and hard tissues that support the tooth (the periodontium), leading to tooth loss [1]. Standard periodontal treatments, as well as in endodontics, rely on the chemo-mechanical debridement and disinfection of the infected area. Where and when possible, the remaining defects are remedied with autologous or synthetic materials. The soft and hard tissues’ full regenerative potential is usually limited and unpredictable due to different reasons; after the periodontal treatment, there is an open wound left behind, exposed to the harsh oral environment and aggressive bacteria [144,145]. Patient compliance and habits (oral hygiene, smoking, diet, drugs, and medications, etc.) pose a big variable, as well as patient systemic conditions that influence the overall response to the infections (immunocompromising diseases, diabetes, etc.). The development of MSC regenerative dental treatments could help to rebuild lost tissues and prevent further damage to the periodontium. The idea is the same as in endodontics (placing an engineered scaffold bearing stem cells and signaling factors into a defect, expecting a consequential total regeneration of the defect) [146]. Results from several studies testing the MSC perio-concepts are probably even more variable than those from endodontic studies, showing moderate or even no advantage in comparison to traditional treatments, so larger steps in this field of dentistry are yet to be made [147,148].

### 4.5. Temporomandibular Joint Disorders

#### 4.5.1. Cell-Based Therapies

TMJ is a ginglymoarthrodial joint that connects the condyle of the mandible with glenoid fossa of the temporal bone [149]. The joint itself is divided into superior and inferior parts by a fibrocartilaginous disc. It is a key factor in wide range of life-support functions, such as mastication, swallowing, communication, and airway [150]. TMJ is considered as one of the most common joints affected by OA [11,151]. OA affects all tissues within the joints, causing typical cartilage degeneration, inflammation of the synovium, pathological subchondral bone remodeling, and formation of osteophytes [152]. These pathophysiological events lead to clinical symptoms, such as aberrant crepitations, chronic orofacial pain, and restricted motions of the mandible resulting in functional disabilities [152]. The diagnosis of TMJ osteoarthritis (TMJ-OA) has mainly relied on the clinical evaluation of symptoms followed by radiographic imaging. Recent use of magnetic resonance imaging (MRI) to supplement the clinical findings vastly improved sensitivity and accuracy in the evaluation of TMJ disorders, including TMJ-OA [153]. Disabilities caused by TMJ-OA are becoming a global health problem due to their negative effects on life quality and consequential socioeconomic costs [154]. The management of TMJ-OA encompasses a wide range of procedures that can be divided into conservative and surgical. Conservative treatment strategies include physical therapy, pain, and anti-inflammatory medications, supporting splints, intra-articular injections of corticosteroids, and arthroscopy. Surgical procedures are indicated only in severe cases when conservative methods are not sufficient [6]. Structures of the TMJ have limited self-renewal capacity, and it is precisely the reason why current treatment modalities are mainly symptomatic, without addressing the basic cause of the disease [6]. There is an increasing number of clinical studies on MSCs use in OA therapy of other joints, such as knees, hips, or hands [11,155,156]. MSCs are known for their multidifferentiation potential, thus contributing to tissue regeneration of condylar structures. Furthermore, their evident anti-inflammatory effect enables the application of xenogenic MSCs without inflammatory reaction [157,158,159,160]. Lu et al. [158] concluded that injections of MSCs into the TMJ region reversed cartilage degeneration and pathological subchondral bone remodeling. The regenerative mechanism of MSCs on cartilage is mainly based on increased matrix production and scavenging activity which leads to the conclusion that matrix replenishment is a preliminary factor in cartilage repair [161]. Moreover, MSCs also display immunomodulatory functions by upregulation of anti-inflammatory and downregulation of pro-inflammatory cytokines secretion [162]. Taking into consideration the benefits of cell-based therapy in the treatment of TMJ-OA, there is a potential of revolutionizing current traditional therapeutic options. Further extensive clinical research is required to overcome existing downsides and improve the efficiency of these methods.

#### 4.5.2. Tissue Regeneration Based on Scaffolds for MSCs in TMJ Therapies

Tissue engineering is constantly developing, and there is ongoing progress in scaffold fabrication methods, growth factor delivery, and cellularization strategies. It may provide an improved solution in various areas, such as disc replacement therapies, structurally compromised and disrupted TMJ structures reconstruction, or even as an alternative to total joint replacement. This approach implies a proper combination of cells, scaffolds, and growth factors that cooperate [163]. Scaffolding materials for regeneration require biocompatibility, appropriate mechanical properties, porous structure, long-term stability, and suitable surface chemistry for cell proliferation. The first disc replacements were Teflon-based and unsatisfactory due to negative effects on the condylar structures, such as morphological changes of the condyle, articular disc fibrosis, and large cell body reactions [164,165]. Thus, a long-term stable scaffold with suitable surface chemistry for the replacement of the articular disc is the key factor for the successful treatment of TMD. There are two categories of scaffold materials based on their origin: natural and synthetic. Natural scaffolds used in articular disc regeneration or replacement are made of collagen, chitosan, fibrin, or decellularized extracellular matrix (ECM) sheets. Collagen, the main component of the native articular disc, is a flexible and mechanically unstable material that can be thermally crosslinked to increase its mechanical properties. Collagen scaffold seeded with MSCs has proved to be a suitable material by successfully closing a perforation in the articular disc of a rabbit model [166]. There is also evidence of stable structures with enhanced cell proliferation and ECM deposition formed by the composite scaffold of fibrin gel and lyophilized chitosan [167]. Furthermore, Brown et al. [168] concluded that ECM scaffold derived from porcine urinary bladder provided a suitable inductive template for TMJ disc reconstruction. Synthetic scaffolds do not possess differentiation properties as natural scaffolds do, but they provide superior mechanical properties due to complete control in the fabrication process. At the same time, they remain biocompatible. Synthetic polymers that have been reported as a scaffold for articular discs are polytetrafluoroethylene (PTFE), polylactic acid (PLA), polyglycolic acid (PGA), polylactic-co-glycolic acid (PLGA), and polycaprolactone (PCL) [163]. The initial failure of Teflon as a prosthetic material for TMJ led to an approach where the scaffold was degraded and replaced with the body’s own tissue. This resulted in the development of biodegradable polymers with high initial mechanical properties, which are designed to degrade over time alongside the formation of the new tissue [163,169]. Ahtiainen et al. [170] investigated tissue-engineered bilayer PLA discs as a replacement for the articular disc in rabbit models and obtained no signs of inflammation, infection, or other adverse tissue reactions, but chronic arthrosis and condylar hypertrophy were detected in all of the TMJ joints. Modern technology is also an important factor in the further development and improvement of scaffolds. 3D printing enables a three-dimensional articular disc scaffold fabrication with the incorporation of biomolecules in the desired position. Legemate et al. created 3D printed PCL scaffolds embedded with PLGA microspheres to reconstruct fibrocartilaginous matrix in the articular disc with emphasis on the protection of growth factors encapsulated in the PLGA [171]. These scaffolds were able to release growth factors which resulted in the differentiation of heterogeneous fibrocartilage with chondrogenic, fibrogenic, and osteogenic cells in vitro.

#### 4.5.3. Growth Factor Delivery-Based Tissue Regeneration for TMJ Disorders

Regenerative dentistry relies on the tissue engineering triad in regeneration and/or replacement of tissues in the oral cavity. Growth factors determine the fate of stem or progenitor cells and thus have a vital role in tissue regeneration. The original concept and idea of their use in regenerative medicine was that prepared platelet concentrates, such as PRP, plasma rich in growth factors (PRGF), and platelet-rich fibrin (PRF), were a viable source of autologous growth factors that could then be used to promote and guide tissue healing [172,173]. In a recent study by Brancaccio et al., it was observed that PRF represents a valid alternative to the traditional hemostatic agents, reduces post-operative bleeding, and promotes wound healing [174]. There is also evidence that PRF significantly relieves post-operative pain and swelling and reduces the incidence of alveolar osteitis [175]. The preparation of PRP and PRGF requires bovine thrombin or CaCl_2_ and two stages of centrifugation in order to increase platelet concentration and avoid the incorporation of leukocytes. The procedure for preparing these to concentrate is technically demanding, and recent research has shown that such preparations release growth factors very briefly and contribute very little to regeneration [176,177,178]. Thus, PRF was developed as an improved alternative to PRP with the purpose of avoiding the use of anticoagulants. It is a fibrin matrix rich with platelets and leukocytes, but also with growth factors and cytokines, such as PDGF, VEGF, IL-1β, IL-4, and TGF-β. Fibrin matrix acts as a reservoir of growth factors and cytokines which makes PRF an effective biomaterial [179]. Due to the aforementioned ability to release growth factors and chemotaxis agents involved in tissue repair mechanisms, PRF is increasingly used in the therapy of medication-related osteonecrosis of the jaws (MRONJ) [180,181]. Finally, it is precisely the controlled delivery of multiple active molecules (growth factors) that can improve tissue regeneration because the natural healing process requires upregulation of more than just one growth factor [182].

## 5. Conclusions

Dental tissue-derived MSCs proved to be a precious stem cell source with great therapeutic potential in both oral and systemic diseases. A detailed review of the entire dental stem cells family confirmed their multilineage differentiation capability and valuable therapeutic potential in regenerative medicine. Together with growth factors and/or scaffolds, dental MSCs are making an extremely potent interplay which is of utmost importance in tissue engineering. Following the recent contemporary literature, MSC treatment could benefit major dental branches like endodontics, oral surgery, and periodontics, providing less invasive and more conservative treatment to future patients. Despite a decent number of in vitro and in vivo studies on MSC treatment in regenerative dental medicine, there are still factors that need to be overcome in order to establish more predictable and reliable clinical protocols. In conclusion, further research is necessary to fully understand the pathways and biology of healing processes in regenerative dental medicine, thus making the treatment less expensive and complex.

## Figures and Tables

**Table 1:** Specific characteristics of different dental tissue-derived MSCs.

	Surface Antigens	Immunomodulatory Functions	Differentiation Potential
Dental pulp stem cells (DPSC)	CD13, CD29, CD44, CD59, CD73, CD90, CD105, CD146, STRO-1	release of transforming growth factor beta (TGF-β), prostaglandin E2 (PGE2) and interleukin-6 (IL-6); stimulation of T cells to release TGF-β	odontogenic, angiogenic, myogenic, adipogenic, osteogenic, and neurogenic
Stem cells from exfoliated deciduous teeth (SHED)	CD166, CD146, CD90, CD73, CD29	repression of T helper 17 (Th17) lymphocytes; upregulation of CD206^+^ M2 macrophages	osteogenic, chondrogenic, adipogenic, odontogenic, angiogenic, and neurogenic
Stem cells from apical papilla (SCAP)	CD146, CD90, CD44, CD24, STRO-1	suppression of T cell proliferation	osteogenic, odontogenic, neurogenic, adipogenic, and chondrogenic
Periodontal ligament stem cells (PDLSC)	CD105, CD73, CD44, CD29, CD10	suppression of IL-1β production; suppression of peripheral blood mononuclear cells (PBMNCs) proliferation; downregulation of tumor necrosis factor-α (TNF-α)	chondrogenic, osteogenic, neurogenic, and adipogenic
Alveolar bone-derived mesenchymal stem cells (ABMSC)	CD73, CD90, CD105, STRO-1	immunosuppressive effects on monocyte and T cell activation; secretion of interleukin (IL)-6 and monocyte chemoattractant protein (MCP)-1	osteogenic and adipogenic
Gingival-derived mesenchymal stem cells (GMSC)	CD73, CD90, CD105	upregulation of interleukin-10 (IL-10); suppression of mast cell degranulation; suppression of PBMNCs proliferation	chondrogenic, osteogenic, adipogenic, angiogenic, and neurogenic
Dental follicle stem cells (DFSC)	CD13, CD29, CD44, CD49d, CD56, CD59, CD90, CD105, CD106, CD166, STRO-1	upregulation of TGF-β and IL-6 secretion; suppression of PBMNCs proliferation	odontogenic, cementogenic, and osteogenic
Tooth germ stem cells (TGSC)	CD73, CD90, CD105, CD166	not investigated	osteogenic, adipogenic, chondrogenic, and neurogenic
